# A Subtle Presentation of Pharyngitis and Pneumonia: Lemierre Syndrome

**DOI:** 10.1155/crpu/6371331

**Published:** 2025-04-15

**Authors:** Federico Bellini, Valentino Allocca, Laura Aspidistria, Marco Farinatti, Ippolito Guzzinati, Marta Maria Daniele, Serena Casanova, Francesca Gasparini, Mara Nalin, Sara Saturni, Gian Luca Casoni

**Affiliations:** Respiratory Semi-Intensive Care Unit, Department of Specialistic Medicine, Ulss 5 Polesana, Rovigo, Italy

## Abstract

Lemierre syndrome (LS) is a rare condition with an estimated incidence of 1–10/1,000,000 per year defined as a complication of an oral and nasopharyngeal infection with secondary septicemia leading to septic emboli and internal jugular vein thrombosis. This syndrome was first described by Andre' Lemierre in 1936, before the development of antibiotics. In the preantibiotic era, it was a common condition and it was often characterized by a fatal course within 7–15 days with a mortality rate that could reach up to 80% of cases. After the development of antibiotic therapies, the incidence of LS rapidly declined, and nowadays, it is also known as “the forgotten disease,” but the mortality risk remains high (5%) especially in case of diagnostic delay and inappropriate therapies. We presented a case of a 23-year-old who was referred to our hospital for worsening dyspnea associated with high fever following a pharyngitis in order to raise awareness about this severe rare disease. Long-term outcomes are usually good if proper treatment is started with no delay. The mainstays of treatment for the pulmonary and vascular aspects are antibiotic treatment with or without anticoagulation and chest-tube drainage.

## 1. Introduction

Lemierre syndrome (LS) is a rare condition with an estimated incidence of 1–10/1,000,000 per year defined as a complication of an oral and nasopharyngeal infection with secondary septicemia leading to septic emboli and internal jugular vein thrombosis [1, 2].

*Fusobacterium necrophorum* (57%), *Fusobacterium species* (30%), and *Fusobacterium nucleatum* (3%) are the main commensal Gram-negative bacteria of the oral cavity frequently isolated from blood cultures of patients affected by LS; however, other agents such as *Staphylococcus*, *Streptococcus*, *Proteus*, and *Bacteroides* have been considered [3, 4].

This syndrome was first described by Andre' Lemierre in 1936, before the development of antibiotics. In the preantibiotic era, it was a common condition and it was often characterized by a fatal course within 7–15 days with a mortality rate that could reach up to 80% of cases [5].

After the development of antibiotic therapies, the incidence of LS rapidly declined, and nowadays, it is also known as “the forgotten disease,” but the mortality risk remains high (5%) especially in case of diagnostic delay and inappropriate therapies [5, 6].

LS occurs more commonly in men, mainly among young adults (aged 14–30 years), with a 2:1 male:female ratio described in some studies [6–9].

Patients, otherwise healthy, often complain typical symptoms, such as neck pain, cellulitis, redness, a recent pharyngitis which is then followed by the appearance of fever, tachycardia, tachypnea, hypotension, and a poor saturation (often < 95%) [10–13].

Laboratory findings often show increased inflammation indices such as leukocytosis and elevated C-reactive protein (CRP) and procalcitonin.

Due to the possible presence of septic emboli affecting various organs, it is not uncommon to find other peculiar alterations such as elevated liver enzymes or creatine phosphokinase (signs of organ failure) and elevated platelet count (sign of a prothrombotic condition) [14].

The most important radiological investigations concern the possible presence of pulmonary complications such as lung abscesses associated or not with pleural effusion.

In order to verify the presence of thrombosis, an imaging study of the internal jugular vein is necessary, and for these reasons, radiological tests are usually performed such as chest x-ray and contrast-enhanced chest CT which is often extended to the neck and brain to exclude the presence of jugular thrombosis and septic emboli involving the central nervous system. Once the necessary investigations have been carried out, antibiotic therapy must be promptly started [5, 14].

Carbapenem and piperacillin/tazobactam are commonly used, either as monotherapy or in combination with metronidazole in order to treat infections caused by *Fusobacterium*, *Staphylococcus*, *Streptococcus*, *Proteus*, and *Bacteroides*. The mean duration of antibiotic treatment can range from 10 days up to 8 weeks depending on the evolution of the single clinical case [14].

## 2. Clinical Case

A male patient of 23 years was referred to our hospital for worsening dyspnea associated with high fever following a pharyngitis treated 2 weeks before with antibiotics.

Unremarkable past medical history includes being a social smoker of e-cigarettes, denied recreational drug use, and no high-risk sexual behavior.

Nearly 2 weeks before the hospitalization, the patient reported the onset of sore throat followed by fever and cough with mucopurulent sputum treated, initially, with clarithromycin 500 mg bid and then with ceftriaxone 1 g SID for 10 days followed by levofloxacin 500 mg SID for 6 days with no significant clinical improvement. For the onset of worsening dyspnea associated with high fever, he was referred, therefore, to the emergency department (ED) of our hospital for medical attention.

At admission in the ED, he presented with fever (tympanic temperature 40°C), tachypnea (respiratory rate 30/min), tachycardia (heart rate 120 bpm), and asthenia.

Pulmonary auscultation showed bilateral reduced vesicular murmur associated with bilateral basal crackles. The patient had normal cardiac physical examination and normal abdominal observation.

Arterial blood gas analysis revealed acute hypoxemic respiratory failure, SpO2 85%, that requires oxygen therapy with O_2_ 4 L/min in nasal prongs.

**Figure 1:**
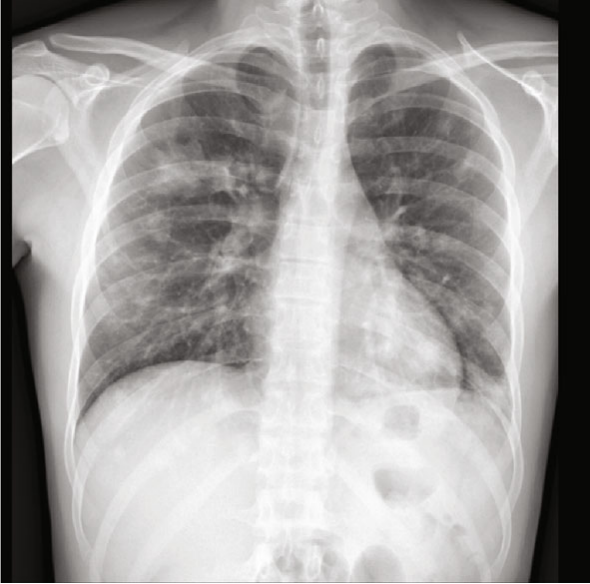
Chest x-ray with bilateral pulmonary consolidations.

**Figure 2:**
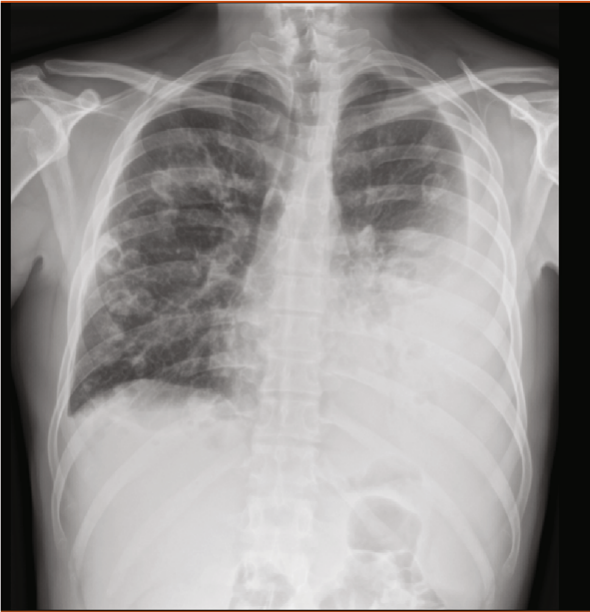
Chest x-ray with bilateral pulmonary nodules and pleural effusion.

**Figure 3:**
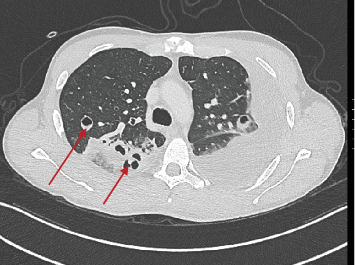
CT scan showing pulmonary excavated nodules with left pleural effusion.

**Figure 4:**
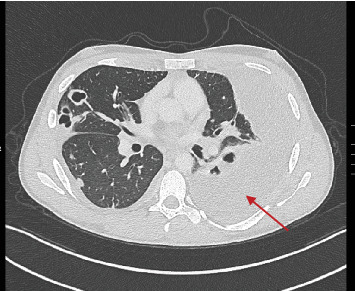
CT scan showing pulmonary excavated nodules with left pleural effusion.

**Figure 5:**
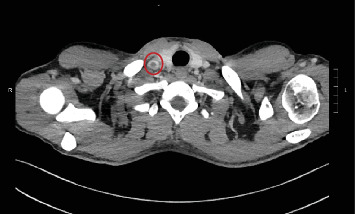
Right internal jugular vein thrombosis (red circle).

**Figure 6:**
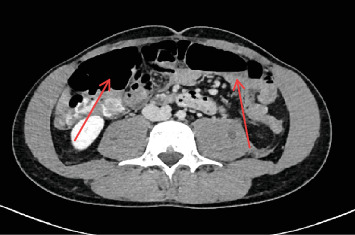
Bilateral microabscess of psoas (red arrows).

Complete blood count showed leukocytosis, piastrinosis, elevated CRP, and elevated procalcitonin (Table 1). Thorax x-ray showed a bilateral pleural effusion associated with bilateral excavated nodule (Figures 1 and 2). A contrast-enhanced CT-was performed and showed bilateral pleural effusion more extensive on the right than on the left side (5.5 vs. 3 cm), multiple bilateral excavated nodules, no sign of pulmonary embolism (Figures 3 and 4). Right internal jugular vein thrombotic apposition was noted (Figures 5 and 6).

The patient was admitted, therefore, in the internal medicine unit and then for the worsening of respiratory failure in our semi-intensive respiratory unit.

The patient was first treated with broad-spectrum intravenous antibiotic therapy with piperacillin tazobactam and linezolid and with low molecular weight heparin (LMWH) BID. For respiratory failure, HFNC (high-flow nasal cannula) therapy was initially used, subsequently replaced by O_2_ at low flows with nasal cannula.

An exploratory thoracentesis was then performed at the level of the left pleural effusion which showed the presence of exudate without the characteristics of a pleural empyema (LDH 1978 U/L, leukocytes 1488 U/mcl, pH 7.0) (Table 1).

Approximately 1100 mL of pleural fluid was initially evacuated, and lung re-expansion was verified with chest x-ray.

**Figure 7:**
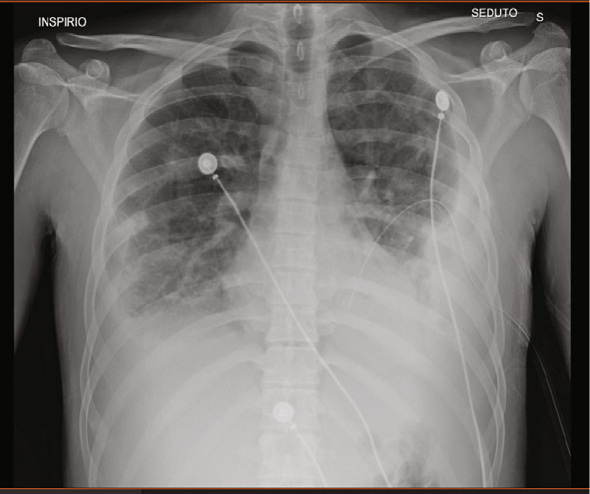
First day of chest drainage placement.

**Figure 8:**
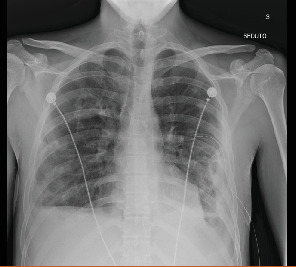
Last day of chest drainage placement.

The following day, given the persistence of abundant pleural effusion, a chest tube (22 Fr) was positioned which allowed the pleural fluid to be completely evacuated in approximately 7 days (Figures 7 and 8).

During the hospital stay, both transthoracic and transesophageal cardiac color Doppler ultrasound was performed which excluded signs of endocarditis.

Laboratory tests highlighted positivity of anticardiolipin antibodies and LAC (lupus anticoagulant) antibodies. However, the search for cryoglobulins and ENA, ANA, ANCA, and rheumatoid factor antibodies was negative.

As regards microbiological investigations, two sets of blood cultures from a peripheral vein, 1 min apart from each other and at the fever peak, were all negative, probably due to the multiple lines of antibiotic therapy performed both before and after hospitalization. Probably for the same reason, the film array respiratory panel assay on a nasopharyngeal swab was also negative.

Urine cultures showed the presence of *Candida albicans*, which was not correlated with the clinical presentation of the patient. Parasitological tests on feces were also negative.

After approximately 10 days of hospitalization, the chest drainage was then removed, and after 20 days, the patient was discharged with indication for a chest x-ray control after 1 month and a jugular venous echo color Doppler (ECD) at 3 months [15].

A visit to the otolaryngologist was performed, and it was recommended to undergo a tonsillectomy and adenoidectomy due to the severe complication of the recent pharyngitis and the endoscopic presence of moderate-sized adenoid remnants.

In follow-up visit at 30 days, thorax x-ray showed a considerable improvement and a stabilization of the results achieved. Pulmonary auscultation did not show pathologic findings. No respiratory symptoms were complained with an actually good quality of life. The ABG in room air at 30 days of follow-up visit was pH 7.50, pO_2_ 75 mmHg, pCO_2_ 37 mmHg, HCO3- 29.2 mmol/L, lac 1.5 mmol/L, SaO2 96.1%.

**Figure 9:**
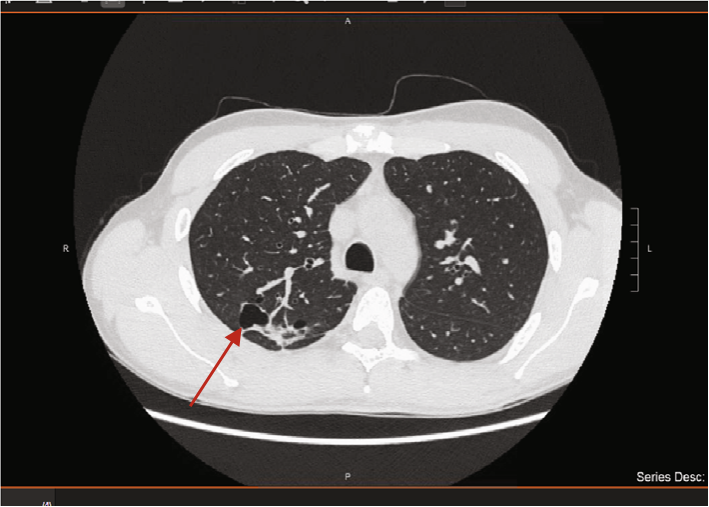
CT thorax at 6-month follow-up: residual small lung cavity at the right upper lobe.

**Figure 10:**
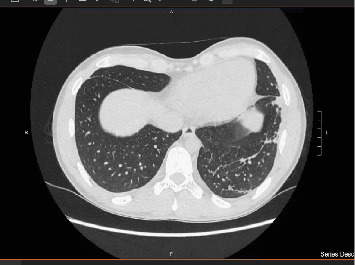
CT thorax at 6-month follow-up.

The CT Thorax performed 6 months after hospitalization showed good resolution of the pulmonary findings with a residual small lung cavity at the right upper lobe and fibrotic interstitial lung abnormalities at the left lower lobe (Figures 9 and 10).

## 3. Discussion

LS is a rare condition but potentially life-threatening if undiagnosed [16, 17], defined as a complication of an oral and nasopharyngeal infection with secondary septicemia leading to septic emboli and internal jugular vein thrombosis. Specific therapies for this disease are still debated. Broad-spectrum antibiotics have been shown to be effective, and the ideal therapy should be based on *β*-lactamase-resistant *β*-lactams, for example, piperacillin–tazobactam. Commonly isolated bacteria (*Fusobacterium* spp.) are intrinsically resistant to macrolide, penicillin, fluoroquinolones, aminoglycosides, and tetracyclines [17]. Our patient was treated with broad-spectrum antibiotics based on piperacillin–tazobactam and linezolid because other agents such as *Staphylococcus aureus* methicillin resistant could be involved in the pathogenesis of this disease [3, 4]. This therapeutic approach is in line with recent reviews in which it is reported that the most frequently used regimen included carbapenem or piperacillin/tazobactam, either as monotherapy or in combination with metronidazole to cover anaerobic organisms. Additionally, the combination of ceftriaxone and metronidazole was noted for its effectiveness against *F. necrophorum* and oral *Streptococci* [14, 18]. Unfortunately, nonspecific bacteria have been isolated from blood cultures, pleural effusion, and a film array respiratory panel assay on nasopharyngeal swab, probably due to a previous domiciliary antibiotic treatment.

Anticoagulation therapy is controversial. Relatively recent metanalyses have proven no statistically significant benefit of anticoagulation therapy on vessel recanalization or mortality [17]. In the work of Allen et al., anticoagulation therapy is considered unnecessary if cerebral sinuses are not interested by the blood. In the most recent literature, the role of anticoagulant use remains debated and the optimal anticoagulation regimens for LS have yet to be clearly defined [18]. Indications for long-term anticoagulation are bilateral or extended clots or signs of poor responsive antibiotic therapy in the first 72 h [5].

Our choice of starting anticoagulation therapy with EBPM was driven by the fact that the patients did not improve in the first days of hospitalization with therapy and for evidence of a large clot that almost completely clogged the internal jugular vein and for evidence of lupus anticoagulant positivity. Subsequently, the patient was treated with edoxaban for 3 months. The follow-up ecocolor Doppler of the supra-aortic trunks showed evidence of complete recanalization of the right jugular vein; therefore, the anticoagulant therapy was then discontinued. Further studies are needed to better understand the optimal therapy in these cases.

Pleural effusion was treated with insertion of chest-tube drainage without fibrinolytic agents in the absence of septation and loculation. There is rapid response of effusion to therapy given no indication to surgery. This approach is in line with other cases present in the literature.

## 4. Conclusion

We presented this case with the aim of enabling early identification and management of LS in order to improve long-term prognosis of the patients. Actually, antibiotic treatment with or without anticoagulation and chest-tube drainage are the main stones to treat the pulmonary and vascular involvement of this condition.

However, more studies are needed on a larger amount of cases to better understand the real epidemiology and pathophysiologic conditions that predispose to these complications.

## Figures and Tables

**Table 1:** Blood tests and pleural fluid tests at admission to our department.

**Laboratory tests (blood samples)**	**Reference values (range)**
Platelets	1,231,000/mcl (v.n.: 150,000–450,000)
Leukocytes	22.030/mcl (v.n. 3600–10,500)
D- dimer	2.91 mcg/mL FEU (v.n. < 0.5)
PCR	24.41 mg/dL (v.n. < 0.5)
Procalcitonin	3.09 ng/mL (v.n. < 0.05)
ALT	152 U/L (v.n. < 55)

**Laboratory tests (pleural fluid)**	**Values (range)**
Aspect	Turbid
Color	Orange
pH	7
Leukocytes	1.488 (< 250 transudate)
Proteins	37 g/L (< 30 transudate)
LDH	1.978 (< 200 transudate)

## Data Availability

The data that support the findings of this study are available on request from the corresponding author. The data are not publicly available due to privacy or ethical restrictions.
